# Autoantibodies against cytokines: phenocopies of primary immunodeficiencies?

**DOI:** 10.1007/s00439-020-02180-0

**Published:** 2020-05-17

**Authors:** Chen-Lung Ku, Chih-Yu Chi, Horst von Bernuth, Rainer Doffinger

**Affiliations:** 1 Graduate Institute of Clinical Medical Sciences, Chang Gung University, Taoyuan City, Taiwan; 2 Department of Nephrology, Chang Gung Memorial Hospital, Taoyuan City, Taiwan; 3 Division of Infectious Diseases, Department of Internal Medicine, China Medical University Hospital, Taichung, Taiwan; 4 School of Medicine, College of Medicine, China Medical University, Taichung, Taiwan; 5 Charité-Universitätsmedizin Berlin, Kinderklinik mit Schwerpunkt Pneumologie, Immunologie und Intensivmedizin, Berlin, Germany; 6 Fachbereich Immunologie, Labor Berlin GmbH, Berlin, Germany; 7 Berlin-Brandenburg Center for Regenerative Therapies, Berlin, Germany; 8 Department of Clinical Biochemistry and Immunology, Addenbrooke’s Hospital, Cambridge, UK; 9 National Institute of Health Research (NIHR), Cambridge Biomedical Research Centre, Cambridge, UK

## Abstract

Anti-cytokine autoantibodies may cause immunodeficiency and have been recently recognized as ‘autoimmune phenocopies of primary immunodeficiencies’ and are found in particular, but not exclusively in adult patients. By blocking the cytokine’s biological function, patients with anti-cytokine autoantibodies may present with a similar clinical phenotype as the related inborn genetic disorders. So far, autoantibodies to interferon (IFN)-γ, GM-CSF, to a group of TH-17 cytokines and to IL-6 have been found to be causative or closely associated with susceptibility to infection. This review compares infectious diseases associated with anti-cytokine autoantibodies with primary immunodeficiencies affecting similar cytokines or related pathways.

## Introduction

Genetically defined, primary immunodeficiencies that impair specific cytokine pathways cause increased susceptibility for selective infectious diseases, and present mostly early in infancy or childhood. Mutations in the cytokine itself, its cognate receptor or downstream signaling molecules may interrupt their biological function, which leads to an impaired immune response. Anti-cytokine autoantibodies were recognized as phenocopies of primary immunodeficiencies (Tangye et al. 2020), and are found in particular, but not exclusively in adult patients. Autoantibodies, produced by auto-reactive B cells, may bind to cytokines. In sufficient concentration, anti-cytokine autoantibodies could block the signaling and neutralize the biological function of target cytokines, by preventing the direct binding to its receptor and (or) depleting the cytokine through forming a cytokine/autoantibodies complex (Piccoli et al. 2015). Autoantibodies against cytokines are, however, not necessarily associated with a respective neutralizing activity (Karner et al. 2016; von Stemann et al. 2017). By blocking the cytokine’s biological function, patients with anti-cytokine autoantibodies may present with a similar clinical phenotype as the related inborn genetic disorders. Although the exact mechanism is largely unknown, the production of autoantibodies may require external exposure to cross-reactive antigens and multiple steps to break tolerance in the adaptive immune response. This may explain why most (but not all) patients with anti-cytokine autoantibodies present later in life. So far, autoantibodies to interferon (IFN)-γ, GM-CSF, to a group of TH-17 cytokines comprising IL-17A, IL-17F, IL-22, IL-23, and to IL-6 have been found to be causative or closely associated with susceptibility to infection. In contrast, high levels of neutralizing autoantibodies may not cause any expected effects in vivo, as, e.g., shown by patients with autoantibodies to type I IFNs (IFNα and IFNω), which do not present with increased susceptibility to viral infections (Weiler et al. 2018). It has been suggested that this may be because of a large number of redundant type I IFN species, resulting in incomplete neutralization of the overall antiviral activity of IFNs by the autoantibodies (Puel et al. 2010).

### Anti-interferon-γ autoantibodies as an etiology in mycobacterial infections in adults

Interferon-γ is a key cytokine produced by activated T cells, natural killer cells, and group I innate lymphoid cells. IFN-γ receptors are expressed widely on most cell types, but especially on myeloid cell (such as macrophages and dendritic cells). The identification of IFN-γ receptor deficiencies (*IFNGR1* and *IFNGR2*) in patients with Mendelian susceptibility to mycobacterial disease (MSMD) demonstrated that IFN-γ plays a critical and non-redundant role in controlling mycobacterial infections (Rosain et al. 2019). Isolated and syndromic MSMD comprises now a group of genetic disorders caused by mutations in 16 published genes (see Table 1), all of them being involved in IFN-γ-mediated immunity, including impaired IFN-γ production (*IL12B, IL12RB1, IL12RB2, IL23R, IRF8, TYK2, ISG15, IKBKG, RORC*) or impaired cellular responses to IFN-γ (*IFNGR1, IFNGR2, JAK1, STAT1, IRF8, CYBB, SPPL2A*) (Bustamante 2020; Martinez-Barricarte et al. 2018; Rosain et al. 2019). More recently, inborn IFN-γ deficiency has been reported in two patients with mycobacterial infection (Kerner et al. 2020). Patients with complete loss of IFN-γ activity present with early onset, severe, disseminated infections caused by low-virulence mycobacteria, such as bacillus Calmette–Guérin (BCG) vaccines and nontuberculous environmental mycobacteria (NTM). Some patients with MSMD may also develop infections with *Mycobacterium tuberculosis*, non-typhoidal *salmonella* (NTS), candidiasis and symptoms of tuberculosis (see Table 1) (Bustamante et al. 2014).

**Table 1:** .

	Primary immunodeficiencies					Anti-cytokine autoantibodies			
Cytokine	Gene affected	Protein affected	Leading clinical infectious phenotype	Penetrance	References	Autoantibody target	Leading clinical infectious phenotype	Penetrance	References
IFN-γ	*IFNG*	IFN-γ	Environmental mycobacteria Invasive, non-typhoidal *Salmonella*	complete only in conditions with complete absence of IFN-γ-signaling	Kerner et al. (2020)	anti-IFN-γ	Environmental mycobacteria Invasive, non-typhoidal *Salmonella* Shingles (topical cutaneous VZV reactivation)	probably incomplete, yet not systematically investigated on a population level	Aoki et al. (2018) Browne et al. (2012) Chi et al. (2013) Chi et al. (2016) Chruewkamlow et al. (2016) Doffinger et al. (2004) Hanitsch et al. (2015) Hoflich et al. (2004) Hong et al. (2019) Jutivorakool et al. (2018) Kampmann et al. (2005) Kampitak et al. (2011) Ku et al. (2016) Liew et al. (2019) O'Connell et al. 2014 Patel et al. (2005) van de Vosse et al. (2017) Wipasa et al. (2018); Wongkulab et al. (2013); Wu et al. (2018) Wu et al. (2020) Xie et al. (2016)
	*IFNGR1*	IFN-γ-receptor-1			Jouanguy et al. (1996) Jouanguy et al. (1997) Jouanguy et al. (1999) Newport et al. (1996)				
	*IFNGR2*	IFN-γ-receptor-2			Doffinger et al. (2000) Dorman & Holland (1998) Rosenzweig et al. (2004) Vogt et al. (2008)				
	*IRF8*	IRF8			Hambleton et al. (2011)				
	*SPPL2A*	SPPL2a			Kong et al. (2018)				
	*CYBB*	gp91^phox^			Bustamante et al. (2011)				
	*ISG15*	ISG15			Bogunovic et al. (2012)				
	*JAK1*	JAK1	Environmental mycobacteria Invasive, non-typhoidal *Salmonella* viral infections		Eletto et al. (2016)				
	*STAT1* *(LOF)*	STAT1			Dupuis et al. (2001) Dupuis et al. (2003)				
	*IKBKG*	NEMO			Filipe-Santos et al. (2006)				
	*IL12B*	IL12-p40	Environmental mycobacteria Invasive, non-typhoidal *Salmonella* Candidiasis	incomplete	Altare et al. (1998a, b)				
	*IL12RB1*	IL-12-Rceptor-1			Altare et al. (1998a, b) De Jong et al. (1998)				
	*IL12RB2*	IL-12-receptor-2			Martinez-Barricarte et al (2018)				
	*IL23R*	IL-23R	Environmental mycobacteria	almost complete	Martinez-Barricarte et al (2018)				
	*TYK2*	TYK2			Kreins et al. (2015)				
	*RORC*	RORγ			Okada et al. (2015)				
IL-17A IL-17F	*IL17RA*	IL-17-receptor	Isolated Chronic Mucocutaneous Candidiasis (CMC)	complete	Puel et al. (2011)	anti-IL-17A anti-IL-17F	Chronic Mucocutaneous Candidiasis (CMC)	unknown	Puel et al (2010) Kisand et al. (2010) Burbelo et al., (2010)
	*IL17RC*	IL-17-receptor			Ling et al. (2015)				
	*IL17F*	IL-17		incomplete	Puel et al, (2011)				
	*ACT1*	ACT1		complete	Boisson et al. (2013)				
	*MAPK8*	JNK1	Syndromic CMC connective tissue disease	unknown	Li et al. (2019)				
	*STAT1* (GOF)	STAT1	Syndromic CMC epithelial ulcer/ aphthae	almost complete	Liu et al. (2011) van de Veerdonk et al. (2011)				
	*STAT3* *(LOF)*	STAT3	Syndromic CMC Staphylococcal skin infections	almost complete	Minegishi et al. 2007 Holland et al. 2007				
	*ZNF341*	ZNF341			Beziat et al. (2018) Frey-Jakobs et al. (2018)				
	*AIRE*	AIRE	CMC Polyendocrinopathy	almost complete	Nagamine et al. (1997)				
	RORC	RORγ	CMC Environmental mycobacteria	almost complete	Okada et al. (2015)				
GM-CSF	*CSF2RA*	GM-CSF-receptor	Pulmonary alveolar proteinosis Nocardiae	incomplete	Martinez-Moczygemba et al (2008)	anti-GM-CSF	Pulmonary alveolar proteinosis Cerebral *Cryptococcus gattii* infection Nocardiae	unknown	Applen Clancey et al. (2019) CrumCianfione et al. (2017) Kuo et al. (2017) Punatar et al. (2012) Rosen et al. (2013) Rosen et al. (2015) Saijo et al. (2014)
	*CSF2RB*	GM-CSF-receptor			Tanaka et al. (2011)				
IL-6	*IL6RA*	IL-6-receptor	Staphylococcal skin infections	unknown	Spencer et al. (2019)	anti-IL-6	Staphylococcal skin infections Invasive staphylococcal infections Pneumococcal infections	unknown	Bloomfield et al. (2019) Nanki et al. (2013) Puel et al. (2008)
	*IL6ST*	gp130	Staphylococcal skin infections ± craniosynostosis	unknown	Beziat et al. (2020) Schwerd et al. (2017)				
	*STAT3* *(LOF)*	STAT3	CMC Staphylococcal skin infections Pneumococcal infections	almost complete	Minegishi et al. 2007 Holland et al. 2007				
	*ZNF341*	ZNF341	CMC Staphylococcal skin infections	unknown	Beziat et al. (2018) Frey-Jakobs et al. (2018)				
	*MYD88*	MyD88	Invasive pyogenic infections	complete	von Bernuth et al. (2008)				
	*IRAK4*	IRAK4			Picard et al, (2003)				

*GOF* gain-of-function, *LOF* loss-of-function, *CMC* chronic mucocutaneous candidiasis

High titers of highly neutralizing anti-IFN-γ autoantibodies (nAIGAs) were initially reported by several groups in sporadic patients or small case series with NTM infections (Doffinger et al. 2004; Hoflich et al. 2004; Kampmann et al. 2005; Patel et al. 2005). In recent years, however, larger cohorts of nAIGA patients were reported from Southeast Asia, with the majority from Thailand, Hong Kong, Taiwan and Japan (Aoki et al. 2018; Browne et al. 2012; Chi et al. 2013, 2016). Only few of the reported cases did not originate from this region (Hanitsch et al. 2015; Kampmann et al. 2005; O'Connell et al. 2014; van de Vosse et al. 2017). Around 500 patients with nAIGAs have been reported up to now in the literature but the exact prevalence rate of nAIGAs-related disease is unknown (Aoki et al. 2018; Barcenas-Morales et al. 2016, 2019; Browne 2014; Browne et al. 2012; Chi et al. 2013, 2016; Chruewkamlow et al. 2016; Doffinger et al. 2004; Hoflich et al. 2004; Jutivorakool et al. 2018; Kampmann et al. 2005; Patel et al. 2005; Wipasa et al. 2018; Wongkulab et al. 2013; Wu et al. 2018).

Similar to patients with MSMD, mycobacterial infections are the main clinical presentations for patients with nAIGAs, and a considerable proportion of these infections (95%) is severe and disseminated (Aoki et al. 2018; Browne et al. 2012; Chi et al. 2016). Both, slowly-growing and rapidly-growing NTMs, are isolated from patients with nAIGAs, but the distribution of species varies widely and depends on the geographical characteristics (Aoki et al. 2018; Browne et al. 2012; Chi et al. 2016; Wongkulab et al. 2013). Every organ system of the body can be infected by NTM species, but lymph nodes, bones/joints, and lungs are the leading organ systems affected (Aoki et al. 2018; Browne et al. 2012; Chi et al. 2016; Wongkulab et al. 2013). Almost all patients are adults in the range from 40 to 70 years with no sex predominance. However, more recently, two young patients presenting at 10 years and 16 years, respectively, have been reported (Liew et al. 2019). In addition to NTM infections, some nAIGAs patients also were developing Tuberculosis, which may precede, be concomitant with, or follow the diagnosis of NTM infections (Browne et al. 2012; Chi et al. 2016; Kampitak et al. 2011). However, the role of nAIGAs in the pathogenesis of tuberculosis remains to be elucidated.

Invasive NTS is another unique clinical picture among patients with nAIGAs. Among MSMD cases, salmonellosis is found in one-third of patients with IL-12/23-related defects (IL-12Rβ1 deficiency and IL-12p40 deficiency) (Bustamante et al. 2014). In the cases with nAIGAs, a history of salmonellosis is found in 29–40% of patients, which is very similar to the proportion of MSMD patients with IL-12/23 defects (Browne et al. 2012; Chi et al. 2016; Wongkulab et al. 2013). However, it should be noted that salmonellosis was found in < 10% of patients with IFN-γR1 and IFN-γR2 deficiency. It is worth to mention that IL-12-related inborn deficiencies show an increased susceptibility to mycobacterial infections; however, the penetrance is not complete. Anti-IL-12 autoantibodies have been reported in a few patients without mycobacterial infection (Sim et al. 2013), which is consistent with the observation of incomplete penetrance for MSMD in IL-12 pathway-related genetic defects (Fieschi and Casanova 2003). Bacteria other than NTS, such as *Streptococcus* spp., *Burkholderia cepacia*, *B. pseudomallei*, *Staphylococcus aureus*, *Legionella pneumophila*, and *Enterobacteriaceae* are occasionally isolated from the nAIGAs patients (Browne et al. 2012; Chi et al. 2016; Tang et al. 2010).

A variety of viral infections have been reported in patients with nAIGAs, but varicella-zoster virus (VZV) is the major causative agent and presents predominantly as shingles (herpes zoster) among this patient population. Most VZV infections are localized, but occasionally disseminated and severe (Browne et al. 2012; Chi et al. 2013, 2016; Jutivorakool et al. 2018). In contrast to patients with nAIGAs, VZV infections are only sporadically reported in patients with IFN-γR1 and IFN-γR2 deficiency. There is no clear explanation for these differences in the clinical presentations between patients with IFN-γR deficiency and patients with nAIGAs, but the age of disease onset might partially explain the divergent phenotypes between the two patient groups. Patients with complete IFN-γR1 and IFN-γR2 deficiency are children and may have no chance to expose to VZV during their lifetime; therefore, these patients are free from herpes zoster. A few cases with nAIGAs have comorbidities, such as diabetes mellitus and autoimmune diseases; nevertheless, the majority cases have no sign of autoimmune disease other than the nAIGAs (Chi et al. 2016; Doffinger et al. 2004; Hung et al. 2018; Jutivorakool et al. 2018).

No familial nAIGAs cases have been reported so far; however, almost all patients with nAIGAs originate from Southeast Asia and Japan, which suggested that genetic factor(s) might be involved. HLA class I and class II molecular typing showed a strong association of nAIGAs and certain HLA class II molecules in Taiwanese patients (DRB1*16:02 and DQB1*05:02) (Chi et al. 2013) and patients from other Southeast Asian countries, including Thailand, the Philippines, Vietnam, and Laos (DRB1*15:02 and DQB1*05:01) (Ku et al. 2016), with the DR/DQ haplotypes being in close vicinity and, therefore, a strong linkage disequilibrium in both cases. DRB1*16:02 and DRB1*15:02 are commonly found in Southeast Asians, Amerindian and Pacific Islanders, but rarely found in Caucasians and Africans (Gonzalez-Galarza et al. 2011; Middleton et al. 2003). The detailed mechanism of these risk alleles in autoantibodies production is still unknown. HLA II class molecules could present the antigens to CD4 T which guide the B-cell activation and maturation. The associations of DRB1*16:02 and DRB1*15:02 are extremely strong. Therefore, these risk alleles might be directly involved in the pathogenic autoantibodies production. However, as only a very small portion of these risk alleles carriers have developed nAIGAs, other factors, including genetic or environmental factors, are likely to be involved. Their identification will be crucial for our understanding of this disease.

## Antibodies to GM-CSF are associated not only with pulmonary alveolar proteinosis (PAP) but also with extrapulmonary infections and cerebral Cryptococcosis in the absence of PAP

GM-CSF is a haematopoietic growth factor which in particular promotes the development of macrophages, dendritic cells and neutrophils. In the lung, it is important for differentiation and function of alveolar macrophages. High titer, neutralizing autoantibodies against GM-CSF are the autoimmune correlate of the much rarer primary GM-CSF-receptor deficiency causing PAP by impairing the alveolar macrophage-mediated surfactant lipid and protein metabolism leading to accumulation and respiratory insufficiency. In contrast to anti-IFN-γ autoantibodies, there is no association with specific HLA alleles (Anderson et al. 2019).

Patients with autoimmune PAP may present not only with opportunistic infection, e.g., by intracellular pathogens such as *Nocardia* and *Histoplasma* which may be secondary to the underlying lung dysfunction but also with extrapulmonary diseases caused by *Cryptococcus*, *Nocardia* and *Aspergillus* (Punatar et al. 2012; Trapnell et al. 2019). However, it is worth to note that these infections might be secondary to impaired lung function by PAP and/or to therapeutic immuno-suppression.

Recently, anti-GM-CSF autoantibodies have been found in patients with *Nocardia* infection or cerebral *Cryptococcus gattii* infection (Kuo et al. 2017; Rosen et al. 2013, 2015). *Cryptococcus neoformans* is an environmental opportunistic species which causes disease in particular in patients infected by human immunodeficiency virus (HIV). However interestingly, anti-GM-CSF autoantibodies patients mostly suffered from *C. gattii*, but not from *C. neoformans* (Kuo et al. 2017; Rosen et al. 2013; Saijo et al. 2014b). Despite carrying neutralizing autoantibodies to GM-CSF, these patients did not manifest PAP at the time of diagnosis, but PAP developed in only few cases at a later time point (Demir et al. 2018; Punatar et al. 2012; Quah et al. 2018; Rosen et al. 2013). It is possible that in those patients, the autoantibodies may have only dampened the function of GM-CSF to some extent, while still allowing sufficient activity of the alveolar macrophages. Piccoli et al. showed that full neutralization needed the synergistic recognition of non-crossreactive epitopes by multiple anti-GM-CSF clones. This could explain the difference between anti-GM-CSF-positive individuals with and without PAP (Piccoli et al. 2015).

## Autoantibodies against IL-6 predispose to pyogenic infections

Four patients with autoantibodies against IL-6 who developed severe bacterial infections have been published to date (Bloomfield et al. 2019; Nanki et al. 2013; Puel et al. 2008). Further, patients with autoantibodies against IL-6 and severe bacterial infections have been identified (Doffinger and von Bernuth unpublished data). All patients presented with low C-reactive protein (CrP) despite severe pyogenic infections. In these patients anti-IL-6-auto-antibodies were of high titer and neutralized IL-6 (phosphorylation of STAT3 and / or production of CrP). A similar susceptibility for pyogenic infections has been described in patients with impaired production of IL-6 due to defects in MyD88/IRAK/NEMO/IκBα-dependent signaling or with impaired IL-6-signaling due to defects in the IL-6-receptor/gp130/ZNF341/STAT3-dependent pathway (Beziat et al. 2018, 2020; Courtois et al. 2003; Doffinger et al. 2001; Frey-Jakobs et al. 2018; Minegishi et al. 2007; Nahum et al. 2019; Picard et al. 2003, 2010; Schwerd et al. 2017; Spencer et al. 2019; von Bernuth et al. 2008). The thorough comparison of the infectious phenotype in patients with autoantibodies against IL-6, with impaired IL-6 production or with impaired IL-6 signaling reveals specific overlaps: Patients with autoantibodies against IL-6, with impaired IL-6 signaling and with impaired IL-6 production show increased susceptibility for severe pyogenic infections, in particular but not exclusively by staphylococci and pneumococci (Nanki et al. 2013; Picard et al. 2010; Puel et al. 2008); whereas, increased susceptibility to staphylococcal skin infections seems in particular common in patients with autoantibodies against IL-6 and with selectively impaired IL-6 signaling (Nahum et al. 2019; Puel et al. 2008; Spencer et al. 2019).

## Autoantibodies to TH17 cytokines (IL-17A/F, IL-22, IL-23) are associated with chronic mucocutaneous candidiasis

Autoantibodies to Th17 cytokines can be found in patients suffering from type I Autoimmune polyglandular syndrome (APS1) (Bruserud et al. 2016) and thymoma (Wolff et al. 2014). A common denominator between those conditions may be impaired tolerance induction caused by primary (in APS1) or secondary structural (in thymoma) disruption of thymus function (Barcenas-Morales et al. 2016; Cheng and Anderson 2018). In particular, APS1 patients and to a lesser degree patients with thymoma may present with neutralizing autoantibodies to TH17 cytokines including IL-17A/F, IL-22 and IL-12/IL-23, which are associated with CMC (Kisand et al. 2010; Puel et al. 2010). APS1 is a complex auto-immune syndrome with CMC as its only infectious manifestation (Li et al. 2017, 2018). A subset of patients with thymoma, a thymic epithelial cancer, may as well present with CMC which may be in the context of wider infectious complications (Burbelo et al. 2010; Kisand et al. 2010; Rosenberg et al. 2016). The cytokines IL-17A, IL-17F, and IL-22 are mainly produced by Th17 cells and play an important role in the mucosal defense against Candida (Okada et al. 2016). IL-23 is mainly produced by dendritic cells and macrophages and is required for the development of TH17 cells (Gaffen et al. 2014; Langrish et al. 2004). Primary deficiencies in the IL17 axis including IL-17F, IL-17RA, IL17RF and the intracellular adaptor ACT1 have been found to predispose to CMC (Puel 2020). Patients with impaired IL-12/IL-23 signaling show as well an increased incidence of CMC (Okada et al. 2016). Surprisingly, however, patients with IL-23R deficiency were not reported with CMC (Martinez-Barricarte et al. 2018). Furthermore, CMC is part of the infectious spectrum found in syndromic primary deficiencies of CARD9, STAT3, STAT1, RORT, ZNF341, and JNK1 which all show diminished Th17 immunity (Beziat et al. 2018; Frey-Jakobs et al. 2018; Li et al. 2019; Puel 2020).

## Concluding remarks

Up to now autoantibodies to four major groups of cytokines—IFN-γ, GM-CSF, to a group of TH-17 cytokines comprising IL-17A, IL-17F, IL-22, IL-23, and to IL-6—have been found to be causative or closely associated with increased susceptibility to selective infection. The largest body of evidence exists for patients with antibodies against anti-IFN-γ, as more than 500 patients with predominant susceptibility to NTM infections and non-typhoid salmonellosis are described. This clinical phenotype strongly resembles the one of patients with inborn deficiencies of IFN-γ-production or IFN-γ-response. Similarly, multiple patients with autoantibodies against TH-17 cytokines (comprising IL-17A, IL-17F, IL-22, IL-23) whose selective susceptibility to candidiasis strongly resembles inborn deficiencies with impaired production or response to IL-17 were described. Autoantibodies against GM-CSF seem to impair the defense against a rather broad group of pathogens whose common denominator is being controlled by macrophages: *Nocardia*, *Histoplasma*, *Cryptococcus gattii, Aspergillus* and maybe NTM. Autoantibodies to GM-CSF are also the major cause for PAP. Only in this aspect, anti-GM-CSF autoantibodies cause the same disease as inborn defects of the GM-CSF receptor. It is unknown why inborn defects of the GM-CSF receptor do not predispose to infections as do the respective autoantibodies. Autoantibodies against IL-6 have yet been described in only few patients, but seem to predispose to pneumococcal and staphylococcal infections. This selective susceptibility strikingly resembles inborn defects with impaired production of IL-6 or impaired response to IL-6.
